# Neuroblastoma: oncogenic mechanisms and therapeutic exploitation of necroptosis

**DOI:** 10.1038/cddis.2015.354

**Published:** 2015-12-03

**Authors:** S Nicolai, M Pieraccioli, A Peschiaroli, G Melino, G Raschellà

**Affiliations:** 1 Department of Experimental Medicine and Surgery, University of Rome “Tor Vergata”, Via Montpellier 1, Rome 00133, Italy; 2 Institute of Cell Biology and Neurobiology (IBCN), CNR, Via E. Ramarini 32, Rome 00015, Italy; 3 Medical Research Council, Toxicology Unit, Hodgkin Building, Leicester University, Lancaster Road, PO Box 138, Leicester LE1 9HN, UK; 4 ENEA Research Center Casaccia, Laboratory of Biosafety and Risk Assessment, Via Anguillarese, 301, Rome 00123, Italy

## Abstract

Neuroblastoma (NB) is the most common extracranial childhood tumor classified in five stages (1, 2, 3, 4 and 4S), two of which (3 and 4) identify chemotherapy-resistant, highly aggressive disease. High-risk NB frequently displays MYCN amplification, mutations in ALK and ATRX, and genomic rearrangements in TERT genes. These NB subtypes are also characterized by reduced susceptibility to programmed cell death induced by chemotherapeutic drugs. The latter feature is a major cause of failure in the treatment of advanced NB patients. Thus, proper reactivation of apoptosis or of other types of programmed cell death pathways in response to treatment is relevant for the clinical management of aggressive forms of NB. In this short review, we will discuss the most relevant genomic rearrangements that define high-risk NB and the role that destabilization of p53 and p73 can have in NB aggressiveness. In addition, we will propose a strategy to stabilize p53 and p73 by using specific inhibitors of their ubiquitin-dependent degradation. Finally, we will introduce necroptosis as an alternative strategy to kill NB cells and increase tumor immunogenicity.

## Facts

- High-risk NB is resistant to conventional pro-apoptotic therapies.
- MYCN amplification, mutations in ALK and ATRX, and genomic rearrangements in TERT genes are frequent in high-risk NB.
- Although not mutated, p53 and p73 are destabilized in NB.
- Caspase 8 is often compromised in advanced NB stages.
- Necroptosis is an alternative modality of programmed cell death.

## Open Questions

- Are there ongoing clinical trials that exploit specific apoptosis and/or necroptosis defects in NB?
- Is stabilization of p53 and p73 a potentially exploitable way to induce apoptosis/differentiation in NB?
- Is activation of necroptosis an alternative to kill NB cells and to increase their immunogenicity?

In neuroblastoma (NB), several genomic abnormalities have been described and the causative genes of the disease have been searched for.^1, 2, 3^ Some genomic defects such as deletions on chromosomes 1p and 11q or gains on 17q^2, 3^ have been utilized as prognostic markers although the contributing gene(s) whose alteration is responsible for the resulting phenotype, are still unknown. One of the first and doubtlessly most important genetic signature of NB is the amplification of the proto-oncogene *MYCN*.^4, 5, 6^ Amplification leading to aberrant expression of MYCN has been associated with tumor aggressiveness,^7^ resistance to chemotherapy^1^ and inability to differentiate.^8^ NB patients who carry *MYCN* amplification are classified in the high-risk group and their overall survival does not exceed 50% at 5 years from diagnosis.^9^ Nevertheless, there is a significant number of NB patients with poor prognosis whose DNA does not harbor *MYCN* amplification.^1^ The latter observation implies that MYCN is not the only culprit of NB aggressiveness. More recently, activating mutations of ALK were reported in both familial and sporadic cases of neuroblastoma.^10, 11, 12, 13^ In familiar NB, germline mutations in *ALK* gene have been found in ~50% of the cases.^13^ In addition, some sporadic NB acquire somatic mutations of ALK and ~2% display genomic amplification of the gene as reviewed in (ref. 14). ALK is a member of the insulin receptor (IR) superfamily of receptor tyrosine kinases, which shows homology with the leukocyte tyrosine kinase, the insulin-like growth factor-1 receptor kinase and the IR kinase.^14^ In humans, *ALK* is located on chromosome 2p23 and the gene encodes for a single-chain transmembrane protein.^14^ The mutated/amplified full-length ALK leads to cell growth and survival by the activation of the JAK–STAT, PI3K–AKT or RAS–MAPK pathways. In NB, the constitutively activated ALK is complexed with hyperphosphorylated ShcC,^15^ deregulating the MAPK pathway response to growth factors.^16^ Another relevant genetic feature in neuroblastoma is the loss-of-function mutations or deletions of the RNA-helicase ATRX.^17, 18^ In a study of 240 NB cases using a combination of whole-exome, genome and transcriptome sequencing Pugh *et al.*^19^ observed putative loss-of-function ATRX alterations in 9.6% of cases (6 mutations and 17 multi-exon deletions). This study confirmed that alterations of ATRX and MYCN were mutually exclusive and that ATRX alterations were enriched in older children.^17^

**Figure 1:**
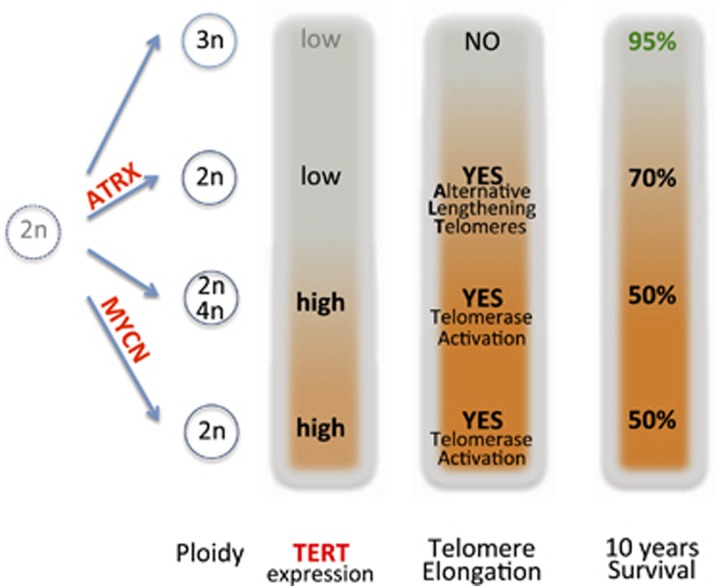
NB-risk subgroups (low and high) inferred from ploidy, ATRX mutations, MYCN amplifications, TERT activation (by genomic rearrangements) and alternative lengthening of telomeres activation

A real breakthrough in the search for genomic alterations that impact on NB aggressiveness comes from the recent observation of telomerase reverse transcriptase (TERT) activation by genetic rearrangements in high-risk NB.^20^ By whole-genomic sequencing of 59 NB cases the authors discovered recurrent genetic rearrangements in the chromosomal region 5p15.33 proximal of TERT. Rearrangements of this region took place only in high-risk NB (12 out of 39=31%). TERT rearrangements, ATRX mutations and MYCN amplifications occurred in a mutually exclusive manner within the high-risk group. The latter observation implies that all these alterations converge on similar effector functions. Of interest, in MYCN-amplified tumors without TERT rearrangements the expression of TERT was nevertheless increased compared with low-risk NB owing to the known function of MYCN as transcriptional activator of TERT.^21^ The expression of TERT was greatly increased in TERT-rearranged NBs compared with the low-risk group. Indeed, rearrangements juxtapose TERT to strong enhancers resulting in a complete epigenetic remodeling of the regulatory region without changes in the gene copy number. The whole-genomic sequencing analysis highlighted also that ATRX mutations, which define another high-risk subgroup, occur only in MYCN-non-amplified and TERT-normal NB, and are associated with alternative lengthening of telomeres (ALT) activity.^20^ This observation suggests that telomere lengthening is a common trait of high-risk NB (i.e., TERT-rearranged, MYCN-amplified and ATRX-mutated tumors) regardless of the mechanism that is utilized for telomere maintenance. Indeed, the most aggressive NB subtypes are characterized by telomerase activation that can derive from TERT rearrangement or MYCN amplification (which activates TERT). In light of these recent findings, we schematize in Figure 1 the different risk subgroups of NB and the genetic aberrations that define each subgroup. A question that arises from the genomic aberrations studies is as follows: which are the pathways and the genes that, following TERT rearrangements or MYCN amplification, become the executioners of the NB aggressiveness? p53, although rarely mutated in NB, is destabilized in MYCN-amplified tumors by the high expression of its ubiquitin ligase MDM2.^22^ Furthermore, p53 pathway is often deranged in NB cells that lack *MYCN* amplification but display telomere lengthening activity.^23^ In the next paragraph we will discuss the role of p53 family and the detrimental effect(s) that its alteration may cause in NB.

The p53 family includes three genes (*p53*, *p63* and *p73*) that have a variety of roles in normal and in transformed cells.^24, 25, 26^ In Table 1 the prominent cellular pathways and principal regulatory circuits that involve p53 family are reported. Similarly to p63, p73 is expressed as several distinct protein isoforms.^27, 28^ In more detail, the usage of two alternative promoters results in the expression of two different N-terminal isoforms: the transcriptionally active p73 (TAp73) proteins, containing a complete N-terminal transactivation domain (TAD), and N-terminally truncated (ΔNp73) isoforms, which lack the TAD and might act as dominant negative molecules by inhibiting the transactivating activity of TAp73 and p53.^25^ Many lines of evidence have clearly demonstrated that TAp73 and ΔNp73 control several biological processes in opposite manner.^29, 30^ Although TAp73 is an inducer of cell cycle arrest and apoptosis, and largely mimics the tumor suppressive activities of p53,^31, 32^ ΔNp73 isoforms promote cancer cell survival and exhibit oncogenic properties.^29^ The phenotypical characterization of selectively deficient mouse models for the N-terminal p73 isoforms confirmed the role of TAp73 and ΔNp73 as tumor suppressor and pro-oncogenic factors, respectively.^33, 34^

Besides their role in controlling tumor growth, p73 isoforms also contribute to the development and differentiation of neuronal tissue. TAp73 null mice show hippocampal dysgenesis with reduction of the neurogenesis in the subgranular zone of the dentate gyrus,^33^ while ΔNp73−/− mice show evidence of neurodegeneration, confirming thus the pro-survival role of this isoforms.^34^ All together, these data indicate that TAp73 and ΔNp73 are important transcription factors whose dysregulation might be an important determinant in tumorigenesis as well as in neuronal development.

## p73 and NB

Alteration of the 1p chromosomal region is commonly observed in NB and the smallest region of overlapping deletions in this region has been refined within 1p36.3.^35^ As p73 maps at 1p36,^28^ it was originally hypothesized that this gene might act as tumor suppressor gene in NB. However, p73 is rarely mutated in primary NB and it is unlikely that it may function as a tumor suppressor in a classic Knudson's manner. Nevertheless, several data indicated that the altered p73 expression rather than its mutation is a determinant factor in the pathogenesis of the NB. The contribution of p73 to NB is indeed thought to depend on the TAp73 to ΔNp73 isoforms' ratio and different molecular mechanisms accounting for altered ΔN : TAp73 expression have been described in NB. Epigenetic modifications, particularly by hyper- or hypo-methylation, are crucial events in cell transformation.^29^ As several human malignancies, such as leukemia and Burkitt's and non-Hodgkin lymphomas, display Trp73 silencing by promoter methylation,^36, 37^ it has been postulated that this type of epigenetic modification could account for the decrease of the expression of the TAp73 isoform observed in NB. However, the analysis of the TAp73 promoter methylation in association with its expression level does not support the idea that the *p73* gene is subjected to genome imprinting in NB.^38^ The idea that the TAp73 activity is associated with NB development is also supported by the role of TAp73 during the neuronal differentiation.^39^ Indeed, one therapeutic approach aimed to restrain NB growth is based on the pro-differentiation action of the retinoic acid.^40^ It has been shown that the expression of the TAp73 isoform is increased during the retinoid-driven NB differentiation and its depletion inhibits differentiation, suggesting that the TAp73 activity is functionally associated with the growth inhibition occurring during the NB differentiation.^39^

In contrast to TAp73, high levels of expression of ΔNp73 have been reported in primary NB.^41^ The increased levels of ΔNp73 observed in NB might functionally inhibit the pro-apoptotic activity of wild-type p53,^42^ and/or physically block the activity of TAp73 allowing the NB to escape from TAp73-driven differentiation program.^39^ In addition, ΔNp73 could inhibit the full activation of ATM and p53, allowing NB to be more resistant to the chemotherapic agents.^34^ Mechanistically, the increased levels of ΔNp73 is likely due to the epigenetic modifications as hypo-methylation of the internal P2 promoter that controls the transcription of this isoform has been observed in NB cell lines and primary tumors.^38, 43^

## N-MYC/MDM2/p53/p73 Axis in NB

As described in the first paragraph, *MYCN* oncogene amplification is one of the most important biological marker of aggressive NB and it occurs in about 20% of primary tumors.^44^ *MYCN* amplification contributes to the NB development and progression by influencing many biological processes, such as cell invasion and motility, cell cycle, immune surveillance, self-renewal and apoptosis.^45^ TP53 mutations are rare in NB at diagnosis,^46^ and amplification of *MYCN* contributes to maintain under surveillance the p53 activity, thought to be its role in the MDM2–p53 pathway.^47, 48^ MDM2 is an E3 ubiquitin ligase that promotes survival by ubiquitinating and driving the degradation of p53. Several tumors, especially those expressing wild-type p53 like NB, are characterized by increased levels of MDM2 expression due to several mechanisms, such as amplification of its locus, increased transcription or increased mRNA or protein stability.^49^ In NB cells it has been shown that MYCN can regulate the MDM2/p53 axis by directly promoting the transcription of MDM2 thus stimulating the ubiquitin-mediated degradation of p53.^22^ Besides p53, MDM2 can also physically interact with TAp73 and as the affinities of MDM2 for p73 are of the same order of magnitude as those for p53, it is likely that these proteins interact in cells, as has been suggested in several studies.^50, 51^ However, MDM2 does not trigger TAp73 proteasome-dependent degradation but rather negatively controls the transcriptional activity of TAp73.^52, 53^ Therefore, by increasing the levels of MDM2, MYCN might not only stimulate p53 protein degradation but also inhibits the TAp73 transcriptional activity, enhancing thus the NB survival and chemo-resistance. It is also worth noting that some data, although controversial, suggest that MYCN can directly affect TAp73 expression levels. MYCN is indeed able to repress the transcription of TAp73 and the reduced expression of p73 correlated with the MYCN overexpression in a statistically significant manner in NB primary tumors.^54^ On the other hand, the overexpression of TAp73 is also able to reduce MYCN expression and thus facilitate the neuronal differentiation program, suggesting an antagonistic role of these two transcription factors on NB cell proliferation and differentiation.^39, 55^ Recently, it has been shown that TAp73 loss determines an increase of the vascularization of lung tumors, suggesting that TAp73 might act as a tumor suppressor by, at least in part, inhibiting tumor angiogenesis. At molecular level, TAp73 stimulates the degradation of the hypoxia-inducible factor-1 alpha (HIF-1*α*) in an oxygen-independent manner.^56, 57^ Interestingly, recent data suggest that ΔNp73 is also involved in tumor angiogenesis. Indeed, upon hypoxia ΔNp73 is stabilized and capable of inducing the expression of *VEGF-A*, the prototypic angiogenic gene.^58^ Similarly to ΔNp73, ΔNp63 is also able to increase the vascular endothelial growth factor (VEGF) secretion by leading to the stabilization of the HIF-1*α* protein.^59^ Therefore, these data suggest a cross talk between the p53 family members and the tumor angiogenesis pathways, potentially involved in the regulation of NB vascularization. Of interest, several data indicated that MYCN is functionally linked with tumor angiogenesis. Indeed, aberrant expression of MYCN had a positive effect on pro-angiogenic factors, including angiogenin and VEGF, and *MYCN* amplification correlates with poor survival, increased dissemination and high vascularization in NB.^45^ In this scenario, MYCN amplification might stimulate tumor vascularization and dissemination by also inhibiting the anti-angiogenic activity of TAp73 either directly or via MDM2.

## Itch as a Potential Therapeutical Target in NB

E3 ubiquitin ligases (E3s) have been shown to have a critical role in regulating cell proliferation, differentiation or apoptosis.^60, 61^ For this reason, the ubiquitin system is often the target of cancer-related deregulation and is critically involved in processes such as oncogenic transformation and tumor progression. Genetic alterations, abnormal expression or dysfunction of E3s is often accompanied by the occurrence of cancer. The HECT-type E3 ubiquitin ligase Itch regulates several important biological processes, such as apoptosis, cell growth and inflammation, and several reports have demonstrated that dysregulation of Itch expression affects the apoptotic response induced by the chemotherapeutic drugs.^60, 61, 62^ Itch depletion by siRNA indeed increases the cytotoxic effect of anti-neoplastic drugs in cancer cell lines and in cancer stem cells.^63^ Furthermore, the *in vivo* administration of siRNA duplex targeting Itch mRNA is effective in sensitizing pancreatic cancer to gemcitabine.^64^ Itch exerts its biological functions mainly by controlling the proteasomal-dependent degradation of a subset of target proteins, including p73. Indeed, among several E3s controlling TAp73 protein levels,^65, 66, 67^ Itch is the most characterized. In detail, in unstressed cells Itch stimulates the proteasome-dependent degradation of TAp73, thereby keeping its expression levels low under normal conditions.^68^ In several tumor cell lines, the induction of TAp73 in response to chemotherapeutic drugs is, at least partially, accomplished through Itch downregulation. We found, in a preliminary analysis, that Itch is expressed in the majority of NB cells tested so far (data not shown). Thus, it is reasonable to hypothesize that in NB cells an Itch-dependent mechanism for negatively controlling TAp73 protein levels might occur and contribute to the chemo-resistance. Thus, targeting Itch ubiquitin ligase activity could be a feasible strategy to stabilize TAp73, enhance its pro-apoptotic activity and sensitize NB cells to the cytotoxic effects of commonly used anti-neoplastic agents. Recently, our laboratory has identified desmethyl-clomipramine (DCMI), the active metabolite of clomipramine, as inhibitor of the Itch autoubiquitylation activity and Itch-dependent ubiquitylation of p73.^69^ Clomipramine is an FDA-approved drug clinically used for the treatment of obsessive compulsive disorders.^70^ Of interest, DCMI increases the cytotoxic activity of conventional chemotherapic drugs in several cancer cell lines as well as in cancer stem cells.^63, 69^ Although it is still not clear whether the DCMI-mediated effect on cancer cell survival completely depends on Itch inhibition, DCMI represents the proof of principle that targeting the E3 ubiquitin ligase Itch might be a novel therapeutical approach to decrease NB cell survival and/or increase the pro-apoptotic effects of conventional anti-neoplastic agents.

## Necroptosis: A Different Modality of Programmed Cell Death

Besides classical caspase-dependent apoptosis, other forms of programmed cell death exist in normal and in transformed cells, which can be activated in response to cellular stress. Necroptosis is a type of necrosis mediated by death receptors (DRs; i.e., Fas, TNFR1/2, TRAIL-R1/2, DR3 and DR6) and their ligands including CD95L (also known as FASL), TNF and TNF-related apoptosis-inducing ligand (TRAIL; also known as TNFSF10), interferons, toll-like receptors, intracellular RNA and DNA sensors, and probably other mediators.^71, 72^ Indeed, another receptor, the transforming growth factor-*β*-activated kinase 1 (TAK1), which is activated through a diverse set of intra- and extracellular stimuli, has been recently added to the list of necroptosis-inducing receptors.^73^

**Figure 2:**
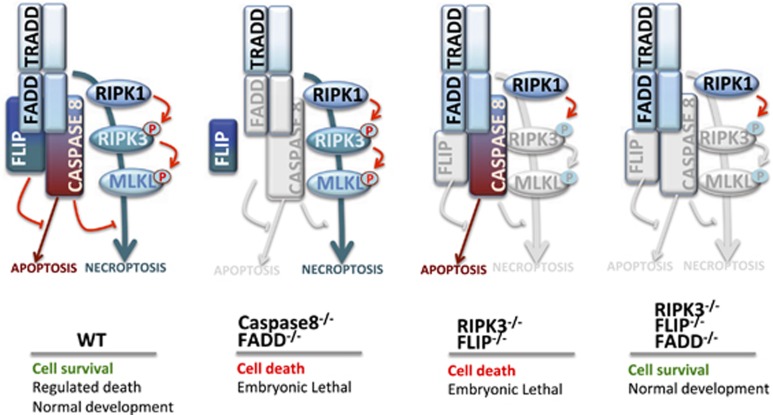
Apoptotic and necroptotic circuitries in wild-type and knockout settings

Seminal work of the laboratory of Jurg Tschopp has defined the role of the first characterized executioner of necroptosis, the receptor-interacting protein kinase 1 (RIPK1).^74^ This original discovery was followed by those of two other essential components of the process, RIPK3^75, 76, 77^ and more recently MLKL.^78^ Necroptosis occurs in the absence of caspase activity and is regulated by the activity of a multi-protein complex called necrosome consisting of RIPK1, RIPK3 and MLKL.^72, 79^ In unstressed, normal conditions, FLIP (in multi-protein complex IIb) inhibits caspase 8 activity, preventing thus apoptosis. At the same time, caspase 8 (in multi-protein complex IIa) prevents the activation of RIPK1 blocking the necroptotic pathway.^71^ The net result of this cross-regulation is survival. Defects in this regulatory circuitry can lead to necroptosis (as in the case of the double knockout of FADD and caspase 8) or to apoptosis (in RIPK3/FLIP double knockout). Of interest, triple knockout of RIPK3, FLIP and FADD rescue the normal phenotype (cell survival). A graphic representation of normal and altered conditions in the apoptosis/necroptosis pathways is reported in Figure 2.

When the necroptotic pathway is unleashed by the engagement of a death receptor, the initial activation of RIPK1 leads to that of RIPK3 by phosphorylation resulting in the recruitment and phosphorylation of MLKL, which causes a conformational change in the pseudokinase domain leading to the exposure of the four-helical bundle domain. Trimerization and movement of active MLKL to the plasma membrane initiates the final step of necroptosis, which terminates with cell rupture and dispersal of the cellular content in the interstitial space.^71^ Trimerization of MLKL requires both RIPK1 and RIPK3 because treating the cells with Necrostatin 1 (Nec-1), a RIPK1 inhibitor, or knocking down RIPK3 prevented the trimerization.^80^

## Necroptosis in Inflammation and Cancer

Inflammation is a main pathologic condition in which necroptosis has an active role. Indeed, the necroptotic process causes a massive release of the so-called damage-associated molecular patterns (DAMPs) from the disintegrating cells. Some DAMP components are active promoters of the inflammatory process that exacerbate inflammation already in place.^81^ In sepsis, a life-threatening condition in which inflammation is a constant feature, necroptosis is associated with increased mortality during TNF-induced systemic inflammatory response syndrome.^82^ The detrimental effect of necroptosis in sepsis is blocked by the presence of caspase 8, which promotes RIPK1 and/or RIPK3 cleavage and inhibits necroptosis.^83^ On the contrary, in cancer therapy, exploitation of necroptotic cell death may open novel avenues for the treatment of apoptosis-resistant tumors. Cancer cells are known to shift from classical apoptosis to other forms of cell death such as autophagy, pyroptosis and necroptosis, some of which entail immunogenicity after anticancer treatments.^84^ It is also well recognized that therapy-resistant cancer stem cells (CSCs) have a higher antiapoptotic activity than that of their counterparts.^85^ Therefore, it would be extremely useful to exploit necroptosis induction in cancer cells for CSC-directed therapeutic application but also the resultant immunogenicity to modulate antitumor immunity.^84^ The latter observation is extremely important in light of the recent advances and applications of immunotherapy in cancer.^86^

## Necroptosis Induction in NB: A Route to Novel Therapies?

**Figure 3:**
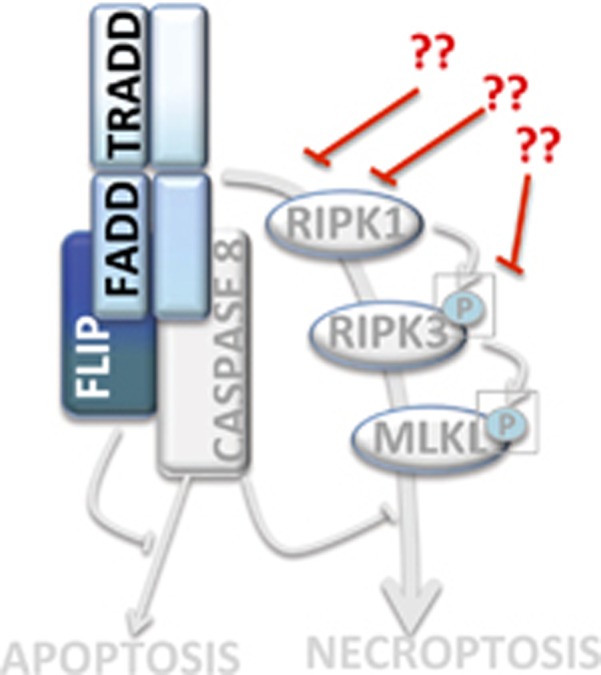
Apoptosis and necroptosis defects in NB. Caspase 8 is often defective in high-risk NB, or its pro-apoptotic activity can be blocked by FLIP. The potential points at which the necroptotic circuitry is interrupted are shown in red

**Figure 4:**
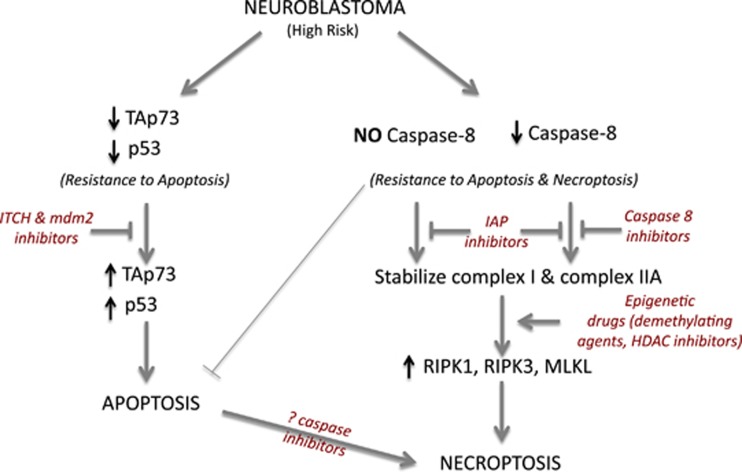
Proposed approaches (in red) to activate apoptotic or necroptotic response in specific subgroups of high-risk NB

In NB the pro-apoptotic activity of caspase 8 is often compromised in advanced stages,^87^ nevertheless these tumors show a marked resistance to death induced by drugs that should trigger necroptosis in a context of caspase 8 deficiency. A scheme pointing out the possible points of deficiency in the necroptotic pathway in NB is depicted in Figure 3. Extensive experimental evidence is not available on the proficiency of RIPK1, RIPK3 and other necrosome components in NB. However, preliminary results from our laboratory demonstrated that caspase 8 and necroptosis-associated genes (*RIPK1* and *RIPK3*) are expressed at significantly lower levels in NB cells compared with other tumor cell lines used as controls. Furthermore, *in vitro* tests suggest that several NB cell lines are resistant to necroptosis (SN and MP, unpublished results). As mutations in necroptotic genes have not been described in NB, epigenetic silencing could occur by hypermethylation of the CpG islands located in the regulatory regions of the necroptotic genes and/or by chromatin modifications. Detection of abnormalities in the activity and/or expression of different members of the necroptotic machinery may represent novel useful markers to better define NB aggressiveness and to predict its response to therapy. More importantly, reactivation of the normal function of the necroptotic pathway (e.g., by demethylating drugs and/or HDAC inhibitors) can be a strategy to rescue cell death ability in chemotherapy-resistant NB tumors defective for caspase 8. In Figure 4 are schematized our proposed approaches based on p73/p53-dependent apoptosis and on necroptosis activation with reference to the potential benefits for specific groups of NB patients.

## Concluding Remarks

NB has been a model for geneticists and molecular biologists who classified genetic abnormalities and identified causative genes of the disease.^7, 13, 17, 20^ However, despite intensive research, improvements in clinical outcome of NB have been achieved mostly for low-/intermediate-risk tumors.^3^ Indeed, metastatic NB remains a difficult-to-treat cancer that has benefited relatively little of research advancements. A survey of the ongoing clinical trials (https://clinicaltrials.gov) highlights the coexistence of trials aimed at optimizing existing therapeutic schedules and those that utilize biological/targeted drugs alone or in combination with well-characterized chemotherapeutic drugs. A selection of current clinical trials is reported in Table 2. Few attempts are underway to exploit specific defects in apoptosis and necroptosis of NB cells. In this sense, our proposal outlined in the previous paragraphs, although not yet mature for a therapeutic application, is aimed at steering preclinical and clinical research toward the exploitation of specific pro-apoptotic and pro-necroptotic targets in NB cells minimizing harmful effects in the patients. As a further clue of the importance of genomic variations in NB, Oldridge et al.^187^ have recently reported genomic predisposition to NB mediated by a SNP in a super-enhancer region of the LMO1 gene.

## Figures and Tables

**Table 1:** Prominent pathways and main regulatory circuits that involve p53 family

**Pathway/regulatory circuit**	**References**
Apoptosis	^88, 89, 90, 91, 92, 93, 94^
Cell growth control	^90, 95, 96, 97, 98^
RNA metabolism	^99, 100, 101, 102, 103, 104, 105, 106, 107, 108, 109, 110, 111, 112, 113, 114^
Protein degradation/stability	^67, 69, 115, 116, 117, 118, 119, 120, 121, 122, 123^
Autophagy	^93, 124, 125, 126, 127, 128, 129, 130, 131^
Splicing events	^111, 132, 133, 134, 135, 136^
ROS and cell metabolism	^92, 137, 138, 139, 140, 141, 142, 143, 144, 145, 146, 147^
Chemotherapeutic response	^56, 57, 148, 149, 150, 151, 152, 153, 154^
DNA damage response	^155, 156, 157, 158, 159, 160, 161, 162, 163, 164, 165, 166, 167, 168^
Transcription and translation	^169, 170, 171, 172, 173, 174, 175, 176, 177, 178^
Stemness and lineage determination	^94, 109, 179, 180, 181, 182, 183, 184, 185, 186^

**Table 2:** Current selected clinical trials on NB

**Clinical trials**	**Interventions**	**URL**
124I-Metaiodobenzylguanidine (MIBG) PET/CT Diagnostic Imaging and Dosimetry for Patients With Neuroblastoma: A Pilot Study	Radiation: 124I-MIBG (no-carrier added) Radiation: 124I-MIBG (carrier added)	https://ClinicalTrials.gov/show/NCT01583842
European Low and Intermediate Risk Neuroblastoma Protocol (low and intermediate pediatric NB and neonatal suprarenal masses)	Drug: chemotherapy	https://ClinicalTrials.gov/show/NCT01728155
Phase II Study of Proton Radiation Therapy for Neuroblastoma	Radiation: proton beam radiation therapy	https://ClinicalTrials.gov/show/NCT02112617
Immunomonitoring of Children With Neuroblastoma	Immunological analyses	https://ClinicalTrials.gov/show/NCT01295762
Bivalent Vaccine With Escalating Doses of the Immunological Adjuvant OPT-821, in Combination With Oral *β*-glucan for High-Risk Neuroblastoma	Biological: adjuvant OPT-821 in a vaccine containing two antigens (GD2L and GD3L) covalently linked to KLH	https://ClinicalTrials.gov/show/NCT00911560
Biomarkers in Tumor Tissue Samples From Patients With Newly Diagnosed Neuroblastoma or Ganglioneuroblastoma	Laboratory biomarker analysis; cytology specimen collection procedure	https://ClinicalTrials.gov/show/NCT00904241
Multimodal Molecular Targeted Therapy to Treat Relapsed or Refractory High-risk Neuroblastoma	Drug: dasatinib Drug: rapamycin Drug: irinotecan Drug: temozolomide Drug: irinotecan Drug: temozolomide	https://ClinicalTrials.gov/show/NCT01467986
Study of DNA in Blood Samples From Patients With Neuroblastoma	Laboratory biomarker analysis Genetic: polymerase chain reaction Genetic: polyacrylamide gel electrophoresis Genetic: DNA analysis	https://ClinicalTrials.gov/show/NCT00898391
Monitor Response to Treatment in Neuroblastoma Using 3&Apos;-Deoxy-3&Apos;-Fluorothymidine-Positron Emission Tomography (FLT-PET)	Device: FLT-PET	https://ClinicalTrials.gov/show/NCT01308905
Expanded Access Study of Fenretinide Lym-X-Sorb Plus Ketoconazole in Neuroblastoma	Drug: fenretinide Lym-X-Sorb oral powder Drug: ketoconazole	https://ClinicalTrials.gov/show/NCT02075177
Activated T Cells Armed With GD2 Bispecific Antibody in Children and Young Adults with Neuroblastoma and Osteosarcoma	Biological: IL-2 Biological: GD2Bi-aATC Biological: GM-CSF Other: laboratory evaluations of immune responses	https://ClinicalTrials.gov/show/NCT02100930
Anti-GD2 3F8 Monoclonal Antibody and GM-CSF for High-Risk Neuroblastoma	Biological: anti-GD2 3F8 monoclonal antibody Drug: GM-CSF (granulocyte-macrophage colony-stimulating factor) Drug: oral isotretinoin	https://ClinicalTrials.gov/show/NCT02100930
Fenretinide Lym-X-Sorb+Ketoconazole+Vincristine for Recurrent or Resistant Neuroblastoma	Drug: fenretinide/LXS oral powder Drug: ketoconazole Drug: vincristine	https://ClinicalTrials.gov/show/NCT02163356
Pilot Study of Activated T-Cell Therapy for Refractory/Relapsed Neuroblastoma	Biological: activated T lymphocyte	https://ClinicalTrials.gov/show/NCT01802138
3rd Generation GD-2 Chimeric Antigen Receptor and iCaspase Suicide Safety Switch, Neuroblastoma, GRAIN	Genetic: iC9-GD2 T-cell lymphocytes – frozen cells Genetic: iC9-GD2 T-cell lymphocytes – fresh cells Drug: cyclophosphamide Drug: fludarabine Drug: pembrolizumab	https://ClinicalTrials.gov/show/NCT01822652

All trials above are recruiting and no results are available yet. From www.clinicaltrials.gov

## Abbreviations

NB: Neuroblastoma
TERT: telomerase reverse transcriptase
DAMPs: damage-associated molecular patterns
